# Investigation of Ageing in Bitumen Using Fluorescence Spectrum

**DOI:** 10.3390/ma11081325

**Published:** 2018-07-31

**Authors:** Ning Tang, Yu-Li Yang, Mei-Ling Yu, Wen-Li Wang, Shi-Yue Cao, Qing Wang, Wen-Hao Pan

**Affiliations:** 1 School of Materials Science and Engineering, Shenyang Jianzhu University, Shenyang 110168, China; tangning@sjzu.edu.cn (N.T.); yangyuli1995@163.com (Y.-L.Y.); 15734022236@163.com (M.-L.Y.); 13940347763@163.com (W.-L.W.); 13591599599@163.com (S.-Y.C.); 2 School of Materials Science and Engineering, Northeast University, Shenyang 110918, China

**Keywords:** fluorescence spectrum, bitumen, ageing, parametrization, “blue-shift”

## Abstract

Bitumen ageing is a very complex process and poses a threat to the performance of pavements. In the present work, a fluorescence spectrophotometer was employed to research the change rule of components and the structure of bitumen after the ageing process. The Thin Film Oven Test (TFOT) and Ultraviolet (UV) light treatment were carried out as ageing methods. The properties and components of bitumen were tested before and after aging. The 2D and 3D fluorescence spectra of bitumen were analyzed. The vector of fluorescence peak was calculated for evaluating the ageing process. The results indicated that the ideal concentration of bitumen- tetrachloromethane solution was 0.1 g/L or smaller for avoiding the fluorescence quenching. The coordinates of fluorescent peak appeared “blue-shift” after ageing due to the change of aromatics. In addition, bitumen has already occurred serious ageing when the magnitude of a vector is more than 36.

## 1. Introduction

Bitumen is one of the primary construction materials in road engineering and waterproofing. This means bitumen is exposed to air for a long time and is directly affected by solar radiation, wind, rain and vehicle loads. As is known to many, bitumen easily ages under the actions of the natural environment of heat, light and oxygen, which can result in property degradation of bitumen and, thus greatly shortened life of asphalt pavement [1,2,3]. In order to prolong the life of asphalt pavement, the properties and microstructure of bitumen play a crucial role. Hence, the ageing and anti-ageing process is one of the hot topics in bitumen researches [4,5,6,7,8].

Bitumen ageing is primarily associated with the loss of volatile components, progressive oxidation and ultraviolet radiation during asphalt mixture construction (short-term ageing) and service (long-term ageing). Researchers try to accelerate ageing of bitumen in the laboratory for simulating the bitumen performance in the field [9]. Thin Film Oven Test (TFOT), Rolling Thin Film Oven Test (RTFOT), Pressure Ageing Vessel (PAV) and Ultraviolet light treatment (UV) are methods commonly employed for simulation in the laboratory [10,11,12]. The TFOT and RTFOT are adopted by American Association of State Highway and Transportation Officials (AASHTO) and American Society for Testing Materials (ASTM) as a means of evaluating the ageing of bitumen during plant mixing. The PAV was developed by the Strategic Highway Research Program (SHRP) for simulating the long-term, in-service oxidative ageing of bitumen in the field. The UV method is used to simulate the long-term ageing of bitumen under the radiation in service.

So far, a lot of evaluation methods have been established and carried out to assess the macroscale performance, such as the three conventional properties of bitumen (penetration, softening point, ductility), rheological properties and so on [13,14]. Moreover, spectroscopy has been widely used in bitumen chemistry because it can provide unique fingerprints for the bitumen at molecular (even atomic) level, for example, infrared spectroscopy (IR) to determine the change of molecular functional groups [15,16,17,18,19], chromatography to determine the moving regularity of the average molecular weight and molecular weight distribution curve [20,21,22], and nuclear magnetic resonance (NMR) to analyze the molecular structure and atomic attribution of the molecular structure by hydrogen or carbon spectrum [23,24].

The composition of bitumen is usually divided into four fractions according to similar chemical behavior, including saturates, asphaltenes, resins and aromatics (SARA) [25,26]. Hence, bitumen is a mixture of hydrocarbon compounds. The greater the C/H ratio, the more aromatic rings the bitumen usually contains. The aromatic ring has a flat conjugated ring system. The bonding between atoms is not a discrete single-double bond but is overridden by the π electron cloud [27]. The emergence and development of fluorescence spectroscopic testing techniques provide a new way for investigating the microstructure of bitumen materials. The fluorescence spectrum is based on the analysis of conjugated system compounds, which makes it possible to analyze the ageing process of bitumen by fluorescence spectroscopy [28,29].

In this study, the properties and components of bitumen were tested. The ultraviolet ageing test and thin film oven test in the laboratory were carried out to evaluate the performance of two kinds of bitumen. Furthermore, the proper concentration of the solution of bitumen-tetrachloromethane was confirmed based on the fluorescence quenching phenomenon. A fluorescence spectrophotometer was employed for obtaining the three-dimensional fluorescence spectra. Based on the coordinate of fluorescence spectra and the displacement of the vector, the aging process of bitumen was evaluated.

## 2. Materials and Methods

### 2.1. Raw Materials

PJ-70 and PJ-90 bitumen were obtained from Liaohe Oilfield, Panjin, China. The conventional properties of bitumen are as follows. For PJ-70, the softening point was 49.5 °C; penetration was 72.6 dmm (25 °C); and ductility was 39 cm (10 °C). For PJ-90, the softening point was 46.7 °C; penetration was 86.9 dmm (25 °C); and ductility was 59 cm (10 °C). 

The organic solvent was carbon tetrachloride which was produced by the China Medicine Group (Beijing, China), and the purity was an analytical reagent.

### 2.2. Experimental Methods

● Conventional properties

The conventional properties of bitumen are softening point, penetration and ductility. These properties were evaluated for both neat bitumen and aged bitumen. Following Chinese Standard JTG E20-2011 (China) [30].

● Four components (SARA) analysis

Bitumen was separated into four components (saturates, aromatics, resins, and asphaltenes, SARA) through precipitation and chromatographic column test method. Following Chinese Standard JTG E20-2011 (China) [30].

● Thin film oven test (TFOT)

The thermal oxidation ageing of bitumen was simulated by thin film oven test (TFOT) according to the Chinese standard (JTG E20-2011) [30]. The experimental temperature was 163 °C, ageing time was 5 h.

● Ultraviolet ageing test (UV)

After the TFOT test, 15 g of the aged bitumen was moved into a stainless steel plate with 140 mm in diameter. The thickness of the bitumen film was 1 mm. Afterwards, the plate was placed into the ultraviolet ageing equipment. The UV lamp was a xenon lamp of straight pipe shape with a power of 1800 w. Experimental temperature was 60 °C. The ultraviolet radiation intensity was about 0.5 W/cm^2^, the rain amounts of spray was 8 liters per day, and ageing time was 7 days. 

### 2.3. Fluorescence Spectra

Three-dimensional fluorescence spectrum is one kind of matrix spectrum which is composed of the excitation wavelength (Y), emission wavelength (X) and fluorescence intensity (Z). The data of fluorescence intensity could be obtained when the excitation wavelength and emission wavelength changed. In the present work, a Hitachi F-7000 fluorescence spectrophotometer (made in Tokyo, Japan) was employed for obtaining fluorescence spectrum. 

A 0.01 g of bitumen sample was moved into a test tube. Afterwards, the 20 mL solvent of carbon tetrachloride was added into the test tube with a tight and mild concussion. When the bitumen was completely dissolved, the solution was transferred into a volumetric flask of 50 mL. After 4 h standing the solution can be diluted into a new solution with different concentrations.

For 2D fluorescence spectrum, the emission spectrum was obtained with the fixed excitation wavelength of 260 nm and 360 nm. The scanning range was from 360 to 680 nm, the slit width was 2 nm, and the scanning speed was 2400 nm/min. The obtained data were smoothed due to the noisy influence by using the Savitzky-Golay method.

For 3D fluorescence spectrum, the scanning range of excitation wavelength (EX) and emission wavelength (EM) was from 200 to 800 nm, the slit width of excitation and emission was all 5 nm, and the scanning speed was 2400 nm/min. The original data obtained by the fluorescence spectrophotometer were converted and plotted with MATLAB (2009a, The MathWorks, Natick, MA, USA) for the fingerprint images of bitumen samples.

## 3. Results and Discussion

### 3.1. Performance of Bitumen

**Figure 1:**
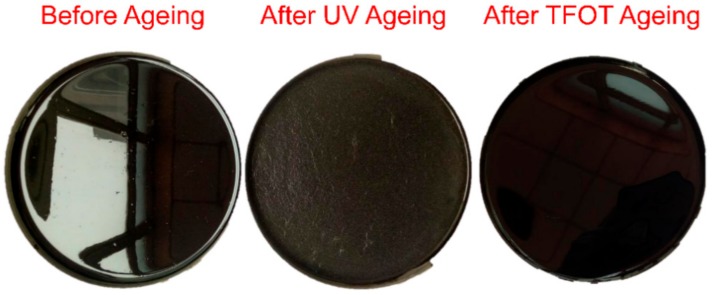
The plate with bitumen before and after ageing.

Figure 1 shows the surface characteristic of bitumen before and after ageing. Before ageing, the neat bitumen had a smooth surface with glossy black. After UV ageing, a lot of wrinkles appeared on the surface like a matt finish. Conversely, there was no significant change after TFOT ageing. It reveals that UV ageing can simulate long-term aging well, and TFOT aging can simulate short-term aging well.

**Figure 2:**
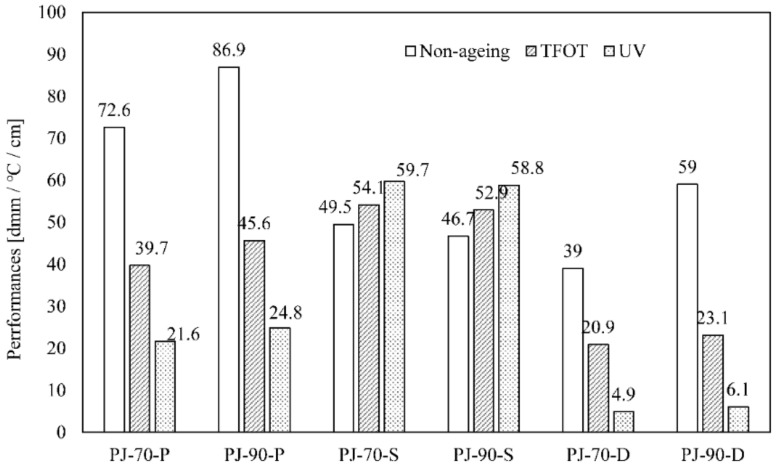
Conventional properties of bitumen before and after ageing.

The performance change of bitumen after ageing was assessed by three conventional properties of bitumen, including softening point, penetration and ductility. The results of softening point, penetration and ductility of PJ-70 and PJ-90 bitumen before and after ageing were shown in Figure 2.

The softening point of PJ-70 bitumen increased after TFOT ageing and UV ageing, with increments of 4.6 °C and 10.2 °C respectively. But, the penetration and ductility decreased more significantly. The residual penetration ratio was 54.7% and 29.8%; ductility retention rate was 53.6% and 12.6%. Changes of the PJ-90 bitumen were similar to PJ-70 bitumen. The softening point increased after ageing, with increments of 6.2 °C and 12.1 °C, respectively. The residual penetration ratio was 52.5% and 28.5%, respectively. The ductility retention rate was 39.2% and 10.3%, respectively. Hence, the bitumen was easy to age through photooxidation, especially softer bitumen (PJ-90). 

### 3.2. Bitumen Fractions

**Figure 3:**
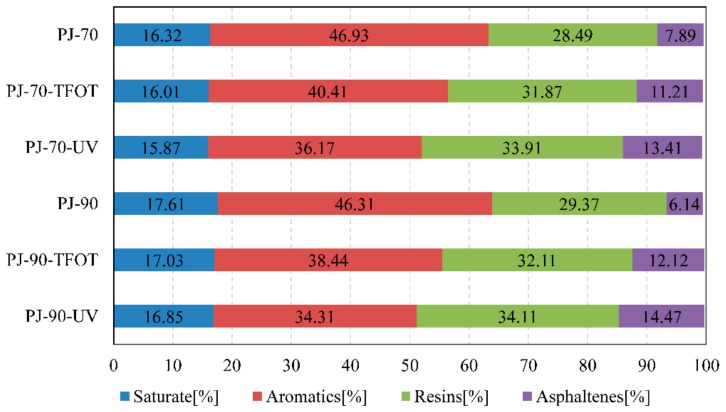
Compositions of bitumen before and after ageing.

Based on the widely accepted analytical approach of SARA, four components were separated from bitumen. The results of composition changes in the two bitumen samples were shown in Figure 3. In general, bitumen kept the same ageing trend as the performance changes after ageing. 

The saturate fraction was nearly unchanged. It illustrated that the chains of saturates were not broken and oxidized during the ageing process. Furthermore, the aromatics are a group of small aromatic naphthenic compounds with low molecular weights. During the ageing process, the aromatics was reduced because it is easy to volatilize. It is also indicated that condensation polymerization had occurred and the chains were broken. At the same time, the oxidation of the chemical functional groups such as carbonyl and sulfoxide resulted in the increase of resins and asphaltenes fraction, and the bitumen became hard and brittle. 

### 3.3. Fluorescence Quenching

A variety of reasons can result in a quenching phenomenon, such as excited state reactions, energy transfer, complex-formation and collisional quenching. As a normal fluorescence fingerprint spectrum which has fluorescent features, the intensity of each data point should be a linear relationship with the concentration of fluorescent components in the bitumen.

Hence, if the fluorescence quenching can be avoided, fluorescent intensity can serve as a proof for contents change of fluorescent material, and the fingerprint picture can be used as a basis for parametrization and image recognition.

Therefore, two kinds of bitumen with different concentrations were tested, and the results are given in Table 1. There was no significant fluorescence quenching in the tests when the concentration of the bitumen diluent was 0.01 g/L, 0.1 g/L or 0.5 g/L. Comparing the different concentrations, the 0.5 g/L is a threshold value and 0.01 g/L is too low to dilute. Thus, the optimized concentration is 0.1 g/L.

### 3.4. 2D Fluorescence Spectrum of Bitumen

**Figure 4:**
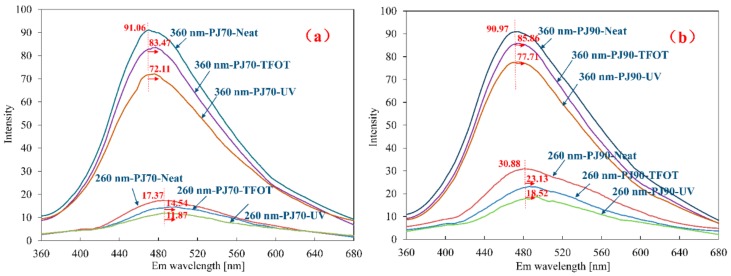
Emission spectrum of bitumen: (**a**) PJ-70 and (**b**) PJ-90.

In order to obtain the emission spectrum of bitumen, two different excitation wavelengths were employed, 260 nm and 360 nm, respectively. A wavelength of 260 nm was commonly used for distinguishing the amounts of benzene ring in the aromatics. In addition, it was also the maximum absorption wavelength when using UV/VIS (ultraviolet and visible) spectrometer. For 360 nm, it was a commonly used excitation wavelength for petroleum. The results are shown in Figure 4.

For both neat bitumen samples, there was little or no difference for parabolic shape or intensity. However, a contradictory phenomenon appeared after bitumen ageing. The intensity of bitumen after TFOT ageing was higher than that of bitumen after UV ageing, both for PJ-70 and PJ-90. In fact, this was caused by the fraction changes of bitumen after ageing. As mentioned, the asphaltenes almost cannot glow fluorescent, and the content of asphaltenes fraction increased after bitumen ageing. The more the asphaltenes fraction, the lower the fluorescent intensity. According to the results of composition analysis, the content of asphaltenes fraction just had a good correspondence with the fluorescent intensity. But it was hard to know why the intensity of PJ-90 bitumen was higher than that of PJ-70 bitumen at 260 nm. Whether this variation was derived from fraction changes or due to an experimental error needs to be further elucidated. Furthermore, the peak shifts to a higher wavelength after ageing. It was suspected that the heteroatoms in the asphaltenes made bitumen polarity increase by increasing oxygen content during ageing, and this influenced the physical properties of bitumen.

**Figure 5:**
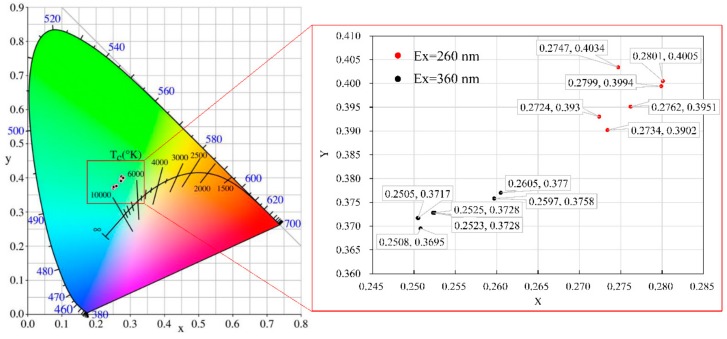
CIE coordinates of bitumen at 260 nm and 360 nm of excitation wavelength.

In addition, the intensity also means the color of light produced by bitumen. The color of light was analyzed by OSRAM ColorCalculator (OSRAM SYLVANIA, Wilmington, MA, USA). The CIE 1931 color space was a quantitative link between wavelengths in the visible spectrum, and colors in human color vision. The results of CIE coordinates were shown in Figure 5.

As shown, bitumen produced a greenish light at 260 nm and a bluish light at 360 nm, respectively. It revealed that the bitumen absorbed more energy at 360 nm due to the intensity of 360 nm of excitation wavelength being higher and holding a higher vibrational frequency for glowing strong light. Moreover, the CIE coordinates were irregular, but not discrete whether different excitation wavelength or ageing treatment.

### 3.5. 3D Fluorescence Spectrum of Bitumen

**Figure 6:**
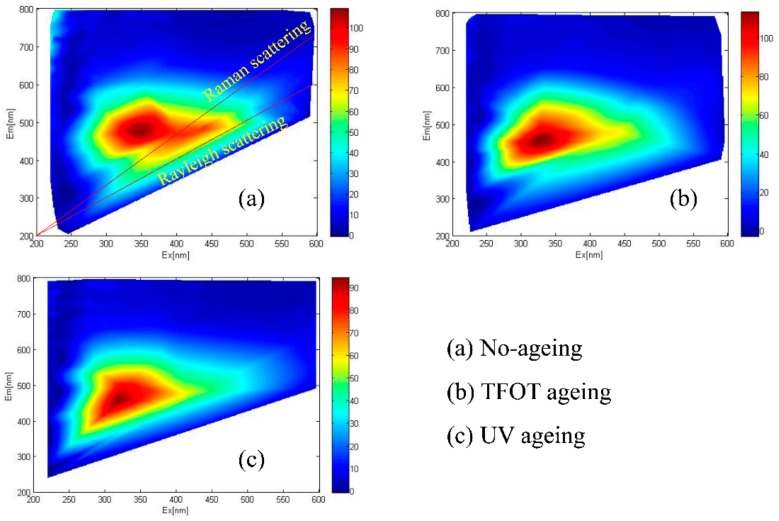
3D fluorescence spectrum of PJ-70 bitumen: (**a**) No-ageing; (**b**) TFOT ageing; (**c**) UV ageing.

**Figure 7:**
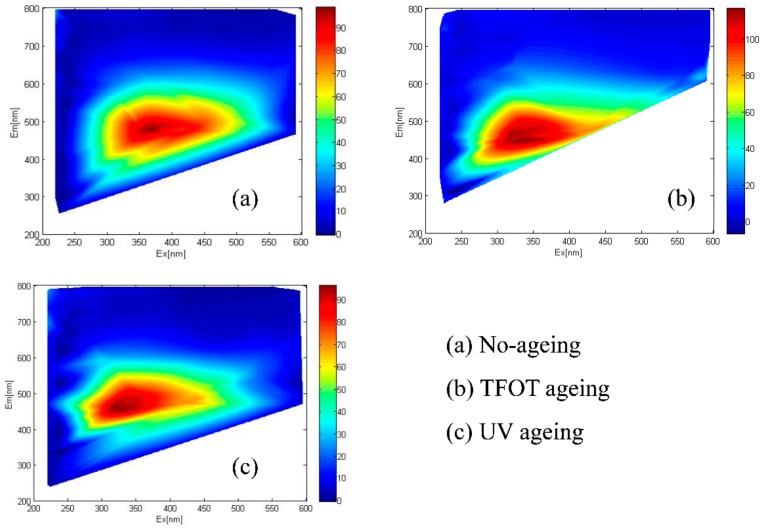
3D fluorescence spectrum of PJ-90 bitumen: (**a**) No-ageing; (**b**) TFOT ageing; (**c**) UV ageing.

Bitumen are derived from petroleum processing, but the composition of bitumen is different due to the different geographical environment and evolution period of petroleum products. Therefore, the three-dimensional fluorescence spectrum of bitumen both has elemental similarities and peak characteristics. Meanwhile, the three-dimensional fluorescence spectrum belongs to the high-dimensional feature space. Irrelevant factors are filtered out through the debasing dimension calculation of the matrix. Thereby, the influence of bitumen on the results of the fluorescence spectrum is reduced to a minimum. The contour images were drawn based on the data of fluorescence spectra of the two bitumen samples, as shown in Figure 6 and Figure 7.

There were two obvious characteristic lines like a wathet blue glow, which was visible rising diagonally up to the right in the above images. For the first line, it was easy to see that the excitation wavelength (EX) of this line was equal to the emission wavelength (EM), the phenomenon is known as “Rayleigh scattering”. For the other one, the EX was bigger than the EM, the phenomenon is known as “Raman scattering”. Two scatterings were produced by the tetrachloride solution of bitumen. When bitumen molecules absorbed lower frequency light energy, the electron only rose to the higher vibrational level in the ground state, rather than being transited to the excited state. Afterwards, the electron returned to the original level in 10^−15^ to 10^−12^ s. If the electron reached the level safely it is Rayleigh scattering. Otherwise, it is Raman scattering.

For TFOT and UV ageing, the intensity of bitumen after UV ageing was more concentrated and more shifted. Furthermore, the intensity area of bitumen after UV ageing was smaller than that of bitumen after TFOT ageing.

Moreover, the intensity peaks of the fluorescence spectrum caused a certain displacement to short wavelength after bitumen ageing, which can be called “blue-shift”. In fact, the fluorescence phenomenon mainly related to the π-conjugate system of fluorescent fraction in the organic matter [31]. If the fluorescent fraction changed after ageing, the maximum absorption wavelength was shifted to the direction of shorter or longer wavelength [27]. Hence, the greater the number of benzene rings, the greater the π electron conjugation degree and the easier the polymer can be excited to produce fluorescence. 

For bitumen ageing, it was dominated by side chain breaking and substituting oxidation [1]. After ageing, the number of benzene rings and the conjugated degree of π electron increased, so the maximum absorption wavelength was supposed to shift to the longer wavelength. In fact, an opposite result was obtained. These fractured alkanes were not easy to expurgate, and continuously accumulated in the internal bitumen molecule so that products overlapped with the benzene rings of bitumen [32,33]. This phenomenon caused the illusion that the number of benzene rings in the large molecular structure of bitumen reduced and suppressed the π electron conjugate. Therefore, the intensity value of the peak dropped to the lower, and the “blue-shift” phenomenon happened. In addition, there was an intensity zone between two characteristic lines but this disappeared after bitumen ageing. It revealed that the fluorescent fraction in the bitumen changed to another non-fluorescent fraction.

### 3.6. Vector of Fluorescence Peak

The coordinates of fluorescence peak (EX,EM) were given in Table 2, which were obtained from Figure 6 and Figure 7 as evaluation parameters for the bitumen ageing process. According to the (EX,EM) coordinates, the computational formula magnitude of vector |AB| was given as Equation (1).

(1) $$\vec{\left|AB\right|}=\sqrt{（x_{2}-x_{1}{）}^{2}+{\left(y_{2}-y_{1}\right)}^{2}}$$

where the peak coordinates of non-ageing bitumen is (x_1_,y_1_), and the peak coordinates of aged bitumen is (x_2_,y_2_). The calculated results were given in Table 2. The magnitude of the coordinate vector was all negative value after bitumen ageing due to the EX and the EM shifting to the direction of short wavelength.

Comparing the two ageing methods, the bitumen after UV ageing was more seriously aged as the magnitude of the coordinate vector was larger. This result corresponded to the changes of conventional performance of bitumen before and after ageing. It revealed that thermal oxidation was prone to ageing of bitumen for the short-term ageing in the laboratory. The aromatics fraction in the bitumen was vaporized causing the deterioration of bitumen. This was accelerated during TFOT due to the high temperature. However, the energy-rich bond of the bitumen could not absorb more energy to fracture the molecule chain during the light oxidation in 7 days [1].

From the view of different bitumen samples, PJ-90 bitumen was easy to age based on the vector calculation and the test results of performance. According to the results of bitumen compositions, the summation of resins and asphaltenes fraction of PJ-70 bitumen were more than that of PJ-90 bitumen, but the aromatics fraction had a slight difference. Furthermore, after the ageing of the thermal oxidation and photooxidation, the saturate fraction remained unchanged, but more resins and asphaltenes were produced. It revealed that the aromatics played a leading role in the ageing process. The side chain was broken and polycondensation of aromatics were more prone to ageing. In other words, the more the resins and asphaltenes fraction, the larger the magnitude of the peak vector and the more serious the bitumen ageing. In addition, the magnitude of peak vector of bitumen after UV ageing was higher than that of bitumen after TFOT ageing. This also matched the experimental results of performance after ageing.

From the view of parametrization, in combination with the conventional performance of the bitumen after ageing, when the magnitude of the vector was more than 36, the bitumen began to pose a threat to performance of the pavement. However, the correlation between the ageing process and the magnitude of the vector is still pending further study.

## 4. Conclusions

It is feasible to have an evaluation of bitumen ageing process by using fluorescence spectrophotometer. Based on the results obtained through the experimental investigation, the following conclusions were obtained. 

The ideal concentration of bitumen-tetrachloromethane solution was 0.1 g/L or smaller for avoiding the fluorescence quenching. The bitumen had a strong energy absorption at 360 nm for producing a bluish light. The other one produced a greenish light at 260 nm.

It can be observed from the fluorescence spectra that the fluorescent intensity of bitumen had a lot to do with the composition of bitumen. Moreover, the peak coordinates shifted to the shorter wavelength because the aromatics had poor stability and were easily oxidized during the ageing process. This displacement was named “blue-shift”. 

The content of aromatics determined the displacement of the fluorescence spectrum peak. The vector of peak coordinates can be calculated and describes the ageing process of bitumen. When the magnitude of the vector was more than 36, it indicated that the bitumen began to pose a threat to the performance of the pavement.

Bitumen is a complicated mixture formed with fractions of different molecular weight, and it can lead to uncertainty about parametrization. Hence, the correlation between the ageing process and the magnitude of the vector is still pending further study.

## Figures and Tables

**Table 1:** Fluorescence quenching of bitumen diluent with different concentrations.

Type	Concentration (g/L)	Peak Intensity	State
PJ-70	0.01	37	Not quenched, characteristic graphics
	0.1	110	Not quenched, characteristic graphics
	0.5	417	Not quenched, characteristic graphics
	1	689	Quenching, shape deformation,
	10	787	Quenched
PJ-90	0.01	33	Not quenched, characteristic graphics
	0.1	114	Not quenched, characteristic graphics
	0.5	458	Not quenched, characteristic graphics
	1	703	Quenching, shape deformation
	10	814	Quenched

**Table 2:** Parametrization of peak coordinate.

Ageing Method		Non-Ageing	TFOT	UV
PJ-70	Coordinate	(358,472)	(332,447)	(318,456)
	Vector	-	(−26,−25)	(−40,−16)
	Magnitude of vector	-	36.07	43.08
PJ-90	Coordinate	(362,478)	(320,458)	(322,451)
	Vector	-	(−32,−20)	(−40,−27)
	Magnitude of vector	-	37.74	48.26
